# [Retracted] Inhibition of EMMPRIN by microRNA-124 suppresses the growth, invasion and tumorigenicity of gliomas

**DOI:** 10.3892/etm.2025.12951

**Published:** 2025-08-18

**Authors:** Yanbin Song, Lei Bai, Feiping Yan, Chen Chen

Exp Ther Med 22:930, 2021; DOI: 10.3892/etm.2021.10362

Following the publication of this paper, it was drawn to the Editors’ attention by a concerned reader that the immunohistochemical data shown in Fig. 1E, the flow cytometric data in Fig. 4A, the TUNEL assay data in Fig. 4C and the tumour images in Fig. 6A were strikingly similar to data appearing in different form in other articles written by different authors at different research institutes that had either already been published elsewhere prior to the submission of this paper to *Experimental and Therapeutic Medicine*, or which were already under consideration for publication (one of which has been retracted). In view of the fact that the abovementioned data had already apparently been published previously, or were under consideration for publication, the Editor of *Experimental and Therapeutic Medicine* has decided that this paper should be retracted from the Journal. The authors were asked for an explanation to account for these concerns, but the Editorial Office did not receive a reply. The Editor apologizes to the readership for any inconvenience caused.

